# The new WHO 2022 and ICC proposals for the classification of myelodysplastic neoplasms. Validation based on the Düsseldorf MDS Registry and proposals for a merged classification

**DOI:** 10.1038/s41375-024-02157-2

**Published:** 2024-01-23

**Authors:** K. Nachtkamp, C. Strupp, M. Vukelja, A. Kasprzak, D. Haase, C. Ganster, B. Hildebrandt, B. Betz, A. Giagounidis, C. Aul, S. Blum, W. K. Hofmann, M. Pfeilstöcker, P. Valent, M. Lübbert, M. Seidl, M. Rudelius, R. Stauder, O. Krieger, K. S. Götze, J. Bobak, A. Kündgen, F. Schulz, S. Dietrich, G. Kobbe, N. Gattermann, U. Germing

**Affiliations:** 1 Department of Hematology, Oncology and Clinical Immunology, Heinrich-Heine University, Düsseldorf, Germany; 2 Department of Hematology and Medical Oncology, Georg August University, Göttingen, Germany; 3 Institute of Human Genetics, Heinrich Heine University, Düsseldorf, Germany; 4 Department of Hematology, Oncology and Palliative Care, Marien Hospital, Duesseldorf, Germany; 5 Department of Hematology, Oncology and Clinical Immunology, Johannes Hospital, Duisburg, Germany; 6 Centre Hospitalier Universitaire Vaudois, Service d’hématologie, Département d’oncologie, and Lausanne University (UNIL), Lausanne, Switzerland; 7 Department of Hematology and Oncology, University Hospital, Mannheim, Germany; 8 Medical Department for Hematology and Oncology, Hanusch Hospital, Vienna, Austria; 9 Ludwig Boltzmann Institute for Hematology and Oncology, Hanusch Hospital and Medical University of Vienna, Vienna, Austria; 10 Department of Internal Medicine I, Division of Hematology and Hemostaseology, Medical University of Vienna, Vienna, Austria; 11 Department of Hematology and Oncology, University of Freiburg Medical Center, Freiburg, Germany; 12 Institute of Pathology, Heinrich Heine University, Düsseldorf, Germany; 13 Department of Internal Medicine, Medical University, Innsbruck, Austria; 14 Elisabethinen Hospital, Linz, Austria; 15 Department of Medicine III, Klinikum rechts der Isar, Technical University of Munich (TUM), Munich, Germany

**Keywords:** Epidemiology, Myelodysplastic syndrome

## To the Editor:

After the French–American–British (FAB) group first described different types of myelodysplastic neoplasms (MDS) in 1982, refined classifications of MDS were proposed by the WHO in 2001, 2008 and 2016, defining minimal diagnostic criteria for MDS [1]. In 2021, a new approach was taken by focusing on genetic aberrations and integrating morphologic features that had not been harnessed previously [2]. In parallel, an international working group independently proposed another refined classification of myeloid neoplasms (international consensus classification, ICC) [3]. The large data base of the Düsseldorf MDS Registry has repeatedly served to validate MDS classifications [4–6]. This we used to validate both classifications in terms of clinical applicability and prognostic impact.

5010 patients in the Düsseldorf MDS Registry diagnosed between 1982 and 2021 as well as 690 patients with acute myeloid leukemia (AML) and myelodysplasia-related changes were used as a comparator for the WHO 2022 category of MDS-IB2. For all patients, central cytomorphological review was performed in our laboratory according to the criteria of the different WHO classifications. It was therefore not difficult to assign cases to the newly proposed categories. In the observation period until Dec 31, 2022, 63% of the patients died, 20.3% developed AML, and 5% were lost to follow-up.

Median age at diagnosis was 71 years (18–104) in the overall study population. Median age was significantly lower in patients diagnosed as MDS with fibrosis (MDS-f) and MDS del(5q). 44% were females. 355 patients (6.2%) were diagnosed as myeloid neoplasm post cytotoxic therapy.

Supplementary Table 1 presents clinical, haematological, and genetic characteristics of the WHO MDS subtypes. The lowest blood cell counts were seen in MDS-f. Remarkably, hematopoietic insufficiency was more pronounced in MDS-f than in MDS-IB1 and MDS-IB2.

Patients with ring sideroblasts (RS) were found in all WHO-defined subtypes but, by definition, mainly in the *SF3B1*-mutated group. The highest percentage of complex karyotypes was detected in MDS-bi*TP53*, followed by MDS-f. *TP53* mutations were, apart from MDS-bi*TP53* patients, also found as monoallelic aberration in all other MDS types, particularly in MDS-f. The highest percentage of peripheral blasts was seen in MDS-bi*TP53* and MDS-f.

There was a clear difference regarding hematopoietic insufficiency and the detectability of chromosomal aberrations between single- and multilineage dysplasia. This difference was also found in patients with RS and/or *SF3B1-*mutation (Supplementary Fig. 1/3A).

Increasing marrow blast percentage and karyotype complexity correlated with the likelihood of AML-progression and the poorest median survival and was highest in MDS-bi*TP53* and MDS-f. Six hundred ninety patients diagnosed as AML-MRC presented with the lowest blood cell counts, the highest percentage of PB blasts, but less complex karyotypes compared to MDS-bi*TP53* and MDS-f.

**Fig. 1:**
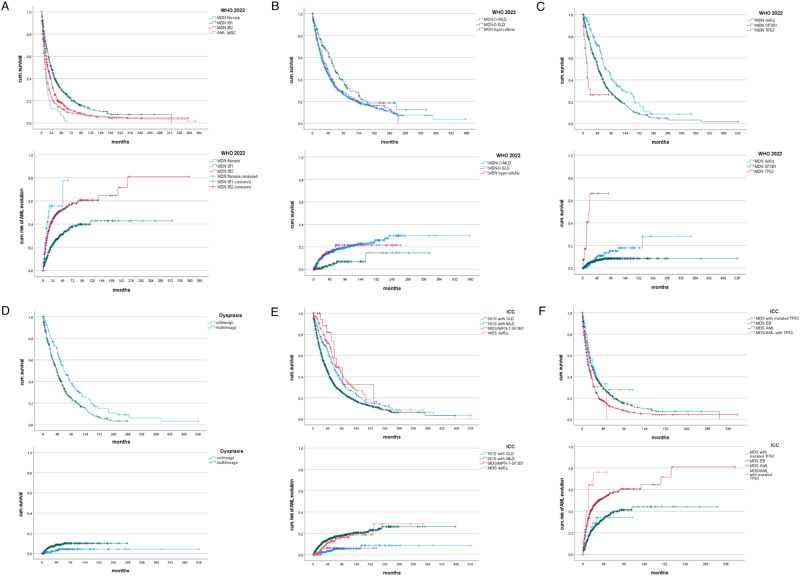
OS and risk of AML evolution of selected MDS subgroups according to WHO 2022 and ICC. **A** WHO 2022: Overall survival and cumulative AML evolution of patients with increased blasts (IB1, IB2, fibrosis) (*p* < 0.0005, *p* < 0.0005). **B** WHO 2022: Overall survival and cumulative AML evolution of patients with low blast count (SLD, MLD, hypocellular MDS) (*p* = 0.006, p0.002). **C** Overall survival and cumulative AML evolution of patients with genetically defined MDS (*p* < 0.00005, <0.00005). **D** Overall survival and cumulative AML evolution of patients with SF3B1 mutation and/or ringsideroblastic phenotype according to lineage dysplasia (*p* < 0.00005, <0.00005). **E** ICC: Overall survival and cumulative AML evolution of patients less than 5% medullary blasts (*p* < 0.00005, <0.00005). **F** ICC: Overall survival and cumulative AML evolution of patients more than 4% medullary blasts (*p* < 0.00005, <0.00005).

Supplementary Table 2 shows the prognostic significance of defining parameters used in WHO 2022 in terms of OS and risk of AML development. Figure 1A–D demonstrates the respective Kaplan–Meier curves. Supplementary Fig. 3A presents further prognostic analyses of MDS types.

Fibrosis grade 2 or 3 was identified in 11% of patients with MDS-IB1/-IB2. Although the database of patients with reliable information on fibrosis was substantially smaller than the entire cohort, the median OS estimates for MDS-IB1 (20 months), MDS-IB2 (17 months), and MDS-f (9 months) were very similar to the respective estimates in the entire cohort.

Multivariate analyses of the prognostic parameters used in the WHO 2022 classification demonstrated, within the restricted database of patients with stringent applicability of WHO and ICC, *TP53* mutational status and multilineage dysplasia were independently associated with poor outcome. We then analysed patients lacking data on fibrosis and cellularity. Again, *TP53* mutation immediately appeared in the regression model, followed by marrow blast percentage and karyotype according to IPSS-R, indicating that these parameters are independently associated with a poor outcome (Supplementary Table 3).

In Supplementary Table 4, clinical, haematological, and genetical characteristics of the different ICC subgroups are given. The del(5q) group was almost identical with the respective WHO type. The *SF3B1* group was significantly smaller, since patients with RS without known *SF3B1* mutation status were allocated to NOS-SLD and NOS-MLD, enlarging those groups. MDS-IB2 according to the WHO 2022 classification corresponded to MDS/AML in the ICC. Patients harbouring a *TP53* mutation were categorized separately. Not surprisingly, these patients more often presented with a complex karyotype. MDS with mutated *TP53* showed the highest cumulative risk of AML development and the poorest survival. Interestingly, median OS and risk of AML did not differ significantly between MDS with mutated *TP53* and MDS/AML with mutated *TP53*. Figure 1E, F presents the Kaplan–Meier curves for OS and AML development.

Supplementary Fig. 2A/B illustrates how patients formerly classified according to WHO 2016 are re-distributed among the new WHO 2022 classification and ICC.

The proposed WHO 2022 classification of MDS requires complex diagnostics but offers a very useful update of morphologically or genetically defined subtypes with prognostic impact. The first definition of a purely molecularly defined MDS type, namely MDS-bi*TP53*, was not triggered by a genotype-phenotype correlation but solely by prognostic considerations, partly due to the highest risk of progression to acute leukemia [7–9]. This genetically defined MDS type is thus clinically justified. To identify these patients, chromosomal analysis is always required, preferentially supplemented by fluorescence in-situ hybridization. Importantly, biallelic *TP53* alteration can only be recognized if next-generation sequencing is performed.

The WHO developed additional morphologically defined MDS types. MDS-f has been introduced, which includes patients from the former MDS-EB1 and -EB2 groups and is characterized by younger age at diagnosis, a preponderance of males, and more pronounced cytopenias. Since hematopoietic insufficiency is severe, prognosis is poor. This has been consistently demonstrated [10, 11] and our own data corroborate these findings. MDS-f can only be diagnosed if a bone marrow biopsy is performed.

This is also mandatory to recognize hypocellularity. Schemenau [12] and Nachtkamp [13] demonstrated that cytomorphology is inferior to histopathological assessment. MDS with hypocellularity has not yet been associated with cytogenetic or molecular features but the clinical observation of a relatively good prognosis and a chance to respond to immunosuppressive therapy justifies recognition as a separate MDS type.

These two histopathological features are not intended as overruling criteria. An overlap of genetically and morphologically defined subtypes is unavoidable. Accordingly, a hierarchy of classification criteria is implicit in the WHO classification. The overruling criterion is biallelic *TP53* mutation, followed by the medullary blast percentage. Next-level criteria in low-blast MDS are del(5q) and *SF3B1* gene mutations. Del(5q) weighs heavier than *SF3B1*, as was the case in 10% of patients with del(5q) in our study population.

While the WHO 2022 classification maintains single- vs. multilineage dysplasia as an optional criterion in MDS with low blast count, this criterion has been abandoned in MDS with *SF3B1* mutation as Malcovati [14] reported this distinction lacked prognostic impact. In contrast, our data indicate prognostic relevance if all patients with ≥15% RS are included, irrespective of *SF3B1* mutation status. In our cohort we had a considerable number of such cases with long-term follow-up. We found that MLD-RS has a significantly worse prognosis because, in contrast to SLD-RS, it includes patients with marked thrombocytopenia and/or granulocytopenia with a higher risk of disease-related complications, but also with a higher risk of AML evolution as shown in Fig. 1D.

Validating the ICC in our patient population, ICC-defined MDS types are also clearly distinguishable in terms of prognosis. The ICC subdivides an MDS/AML group according to *TP53* status, which is comprehensible because of the prognostic impact of *TP53* alterations, particularly if biallelic [9]. The difficult question of where MDS ends and where AML begins is handled by the ICC through introducing MDS/AML (10–19% blasts). However, all larger studies show that MDS-IB2 have a better prognosis than patients with >19% blasts and may indeed include long-term survivors [15]. The term MDS/AML according to the ICC may imply that patients in this group must be treated like patients with AML. We think that this would be misleading and could trigger harmful therapy decisions, thus support maintaining the 20% blast cutoff to define AML.

In our view, a disadvantage of the ICC is its omission of histopathology, namely cellularity and fibrosis. Aforementioned results underscore that they should receive appropriate attention.

**Fig. 2:**
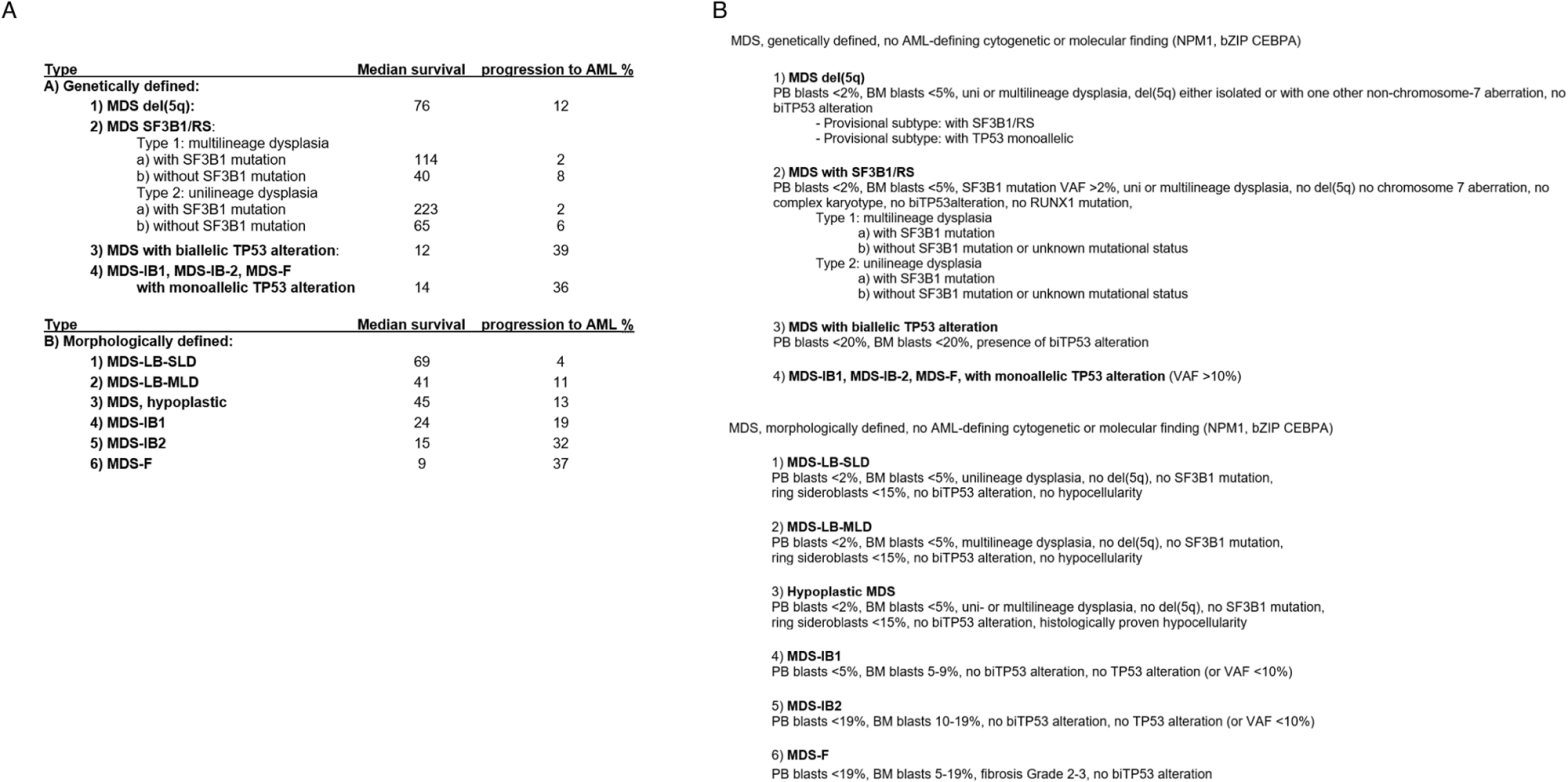
Merged classification for MDS. **A** Median survival and proportion of AML transformation of MDS types according to the merged MDS classification. **B** Proposals for a merged MDS classification.

Dealing with two competing MDS classifications is somewhat cumbersome and complicates the design and comparability of clinical trials. A situation hampering progress in clinical research should be remedied. We would like to suggest how a merger might be possible. A few adaptations regarding the classification criteria may harness the strengths of both classifications as outlined in Fig. 2B. Applying the merged classification to our cohort yielded good separation in terms of OS and risk of AML transformation (Fig. 2A/Supplementary Fig. 3B).

The Düsseldorf MDS Registry demonstrates both classifications categorize genetically and morphologically well-defined subtypes with prognostic relevance. We postulate the WHO 2022 classification offers clearer definitions and, by including histopathology, addresses features of MDS possibly having been underestimated so far and requiring further analysis regarding their molecular causes. Finally, our proposals for a merger may help to develop a re-unified MDS classification that could foster clinical research by facilitating the design and comparability of clinical trials.

### Supplementary information

Supplemental Figure 1

Supplemental Figure 2

Supplemental Figure 3 A-B

Supplemental Table 1

Supplemental Table 2

Supplemental Table 3

Supplemental Table 4
