# Supplementary figures and images for: The new WHO 2022 and ICC proposals for the classification of myelodysplastic neoplasms. Validation based on the Düsseldorf MDS Registry and proposals for a merged classification
Source: Leukemia. 2024 Jan 23;38(2):442–5. doi: 10.1038/s41375-024-02157-2 (PMC10844089; doi:10.1038/s41375-024-02157-2)

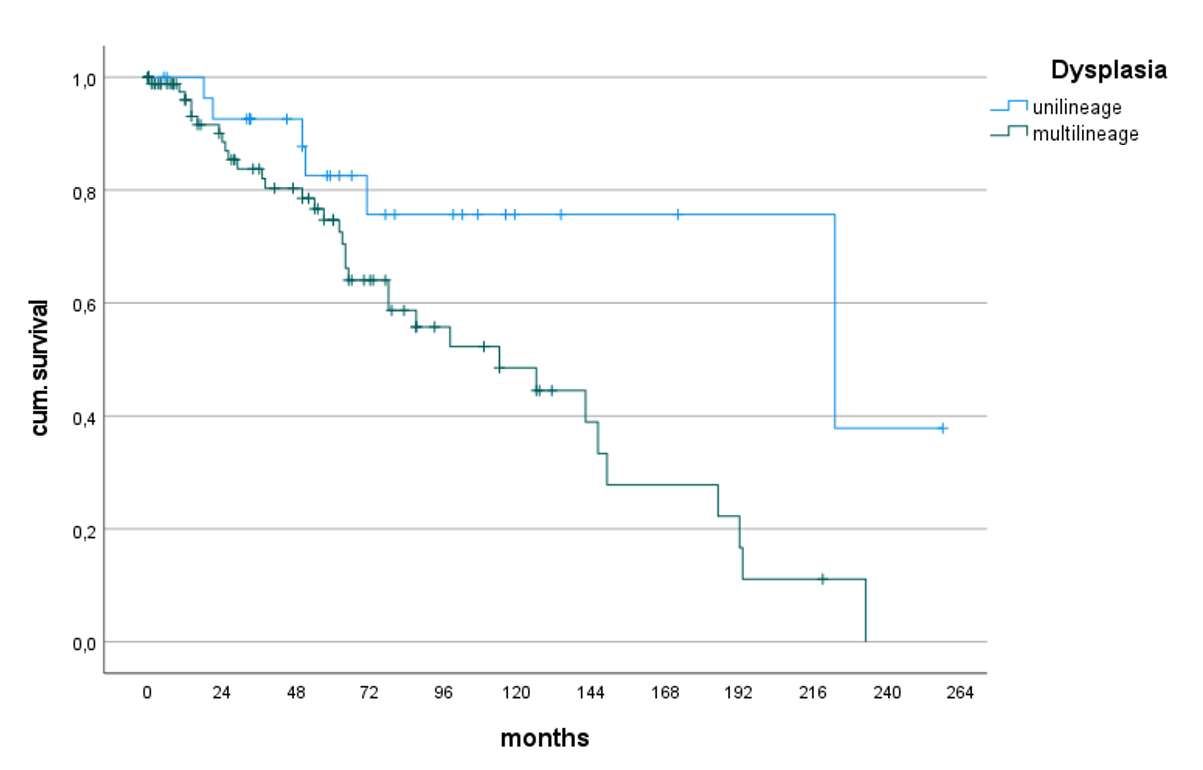

Supplement: Supplementary file 1 — Supplemental Figure 1 [file 41375_2024_2157_MOESM1_ESM.docx]

A


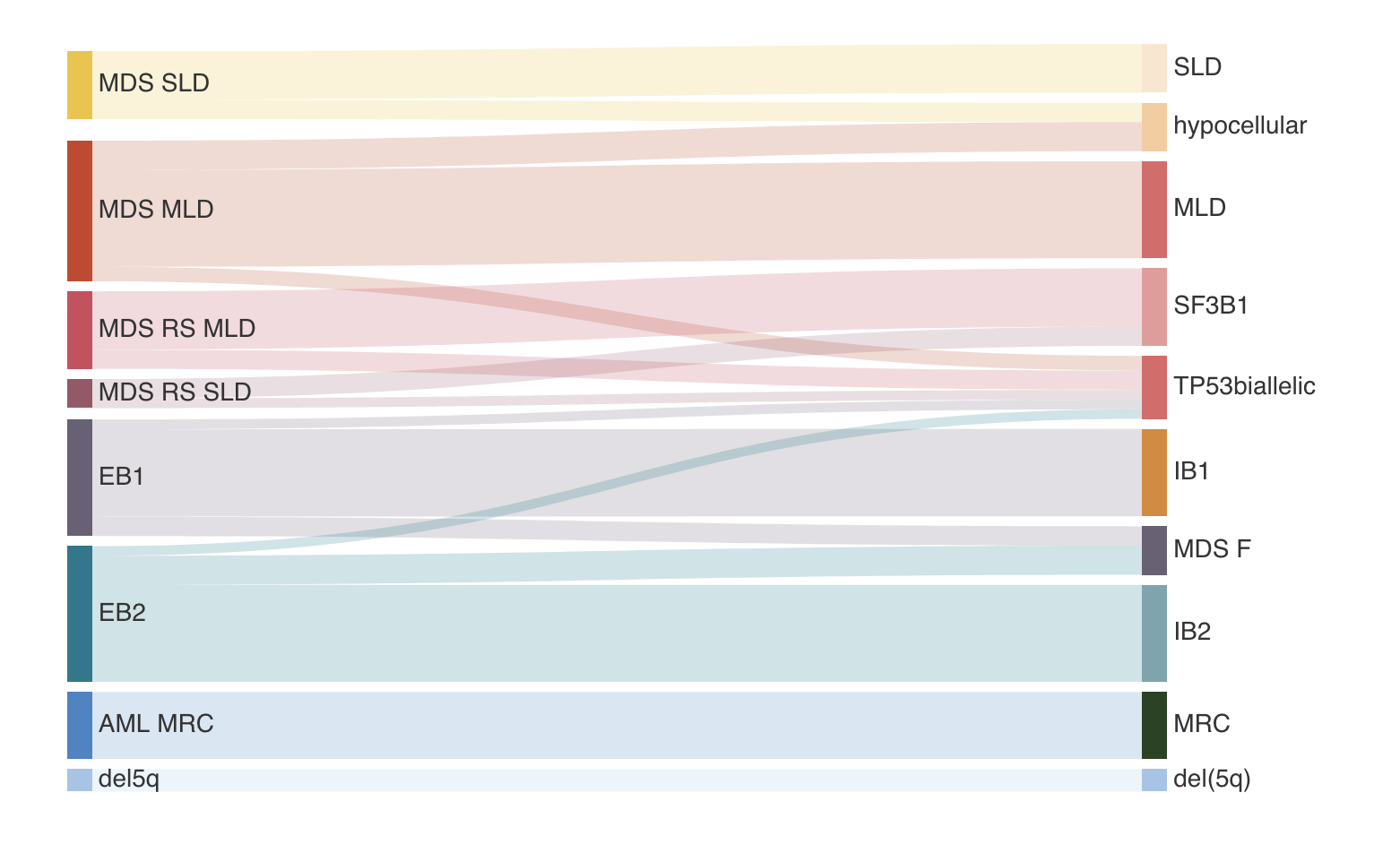


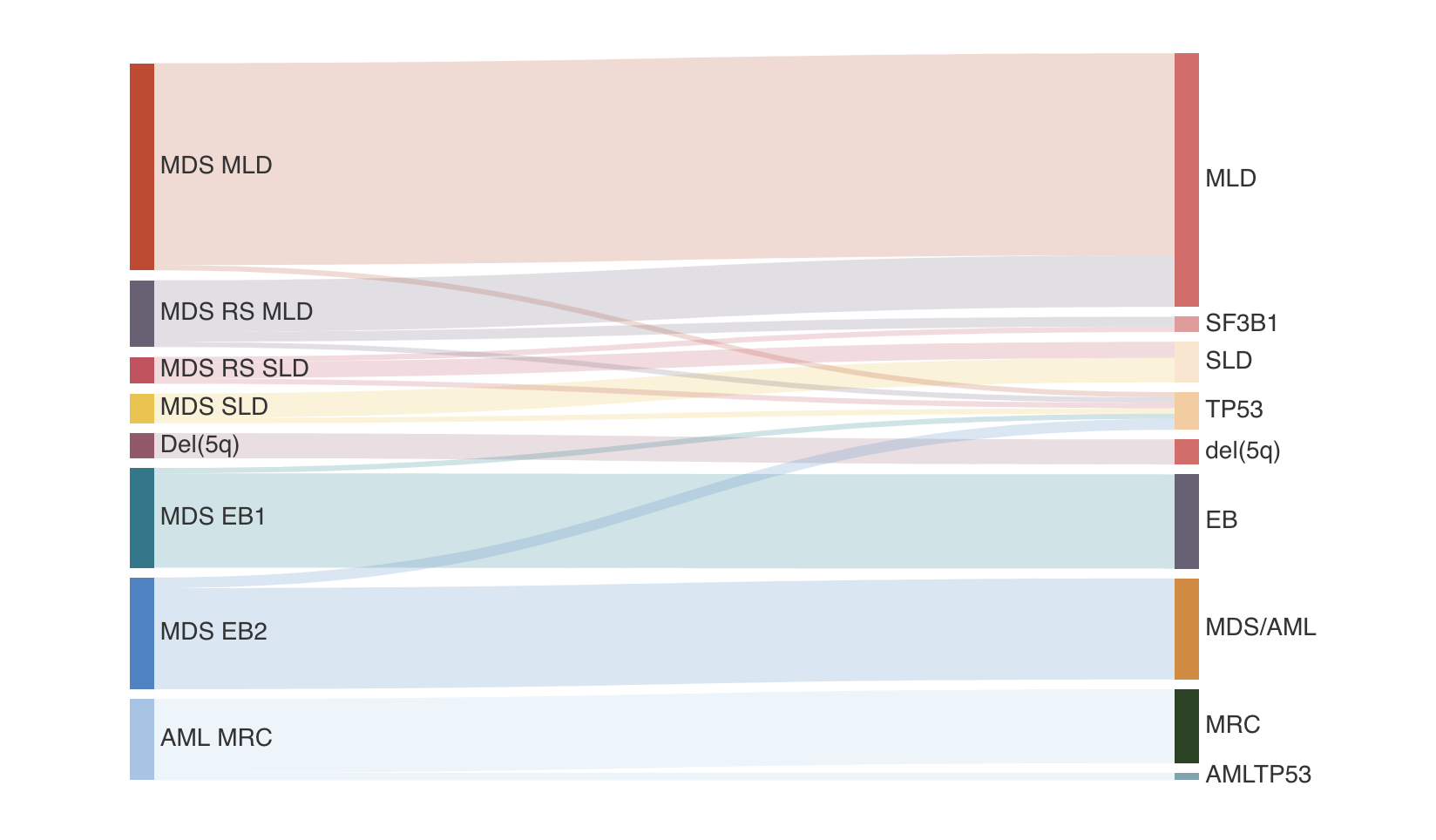
B

Supplement: Supplementary file 2 — Supplemental Figure 2 [file 41375_2024_2157_MOESM2_ESM.docx]
