# Supplementary material for: The new WHO 2022 and ICC proposals for the classification of myelodysplastic neoplasms. Validation based on the Düsseldorf MDS Registry and proposals for a merged classification
Source: Leukemia. 2024 Jan 23;38(2):442–5. doi: 10.1038/s41375-024-02157-2 (PMC10844089; doi:10.1038/s41375-024-02157-2)

A

a) patients with increased medullary blasts and with information on fibrosis (n=548)

Parameter	(n, %)	median survival	χ2	p	Progression to AML (%)	χ2	p
IB1	234 (42.6)	20	28.2	<0.00005	39.4	22.3	<0.00005
IB2	254 (46.4)	17			23.2		
MDS-fibrosis	60 (11)	9			36.7		

b) patients with low blasts and with histological assessment of cellularity (n=691)

Parameter	(n, %)	median survival	χ2	p	Progression to AML (%)	χ2	p
SLD	73 (10.6%)	69	10.2	0.003	2.7	15.2	0.004
MLD	475 (68.7%)	39			9.7		
Hypopl. MDS	143 (20.7%)	50			13.6		

c) patients with low blasts and with banding karyotypes (n= 1558)

Parameter	(n, %)	median survival	χ2	p	Progression to AML (%)	χ2	p
SLD	93 (6%)	70	26.5	<0.00005	3.2	28.9	<0.00005
MLD	917 (58.9%)	42			11.9		
Hypopl. MDS	64 (4.1%)	50			12.5		
SF3B1	259 (16.6%)	63			3.5		
MDS del(5q)	225 (14.4%)	76			10.8		

d) patients with ring sideroblasts and uni- or multilineage dysplasia (n=766)

Parameter	(n, %)	median survival	χ2	p	Progression to AML (%)	χ2	p
RS SLD	176 (24%)	71	17.3	<0.00005	3	4.4	0.03
RS MLD	590 (76%)	47			6		

B

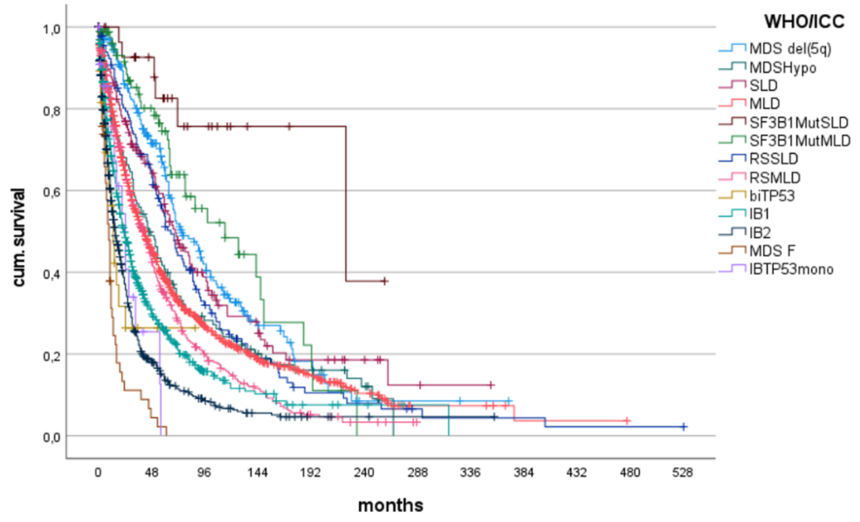

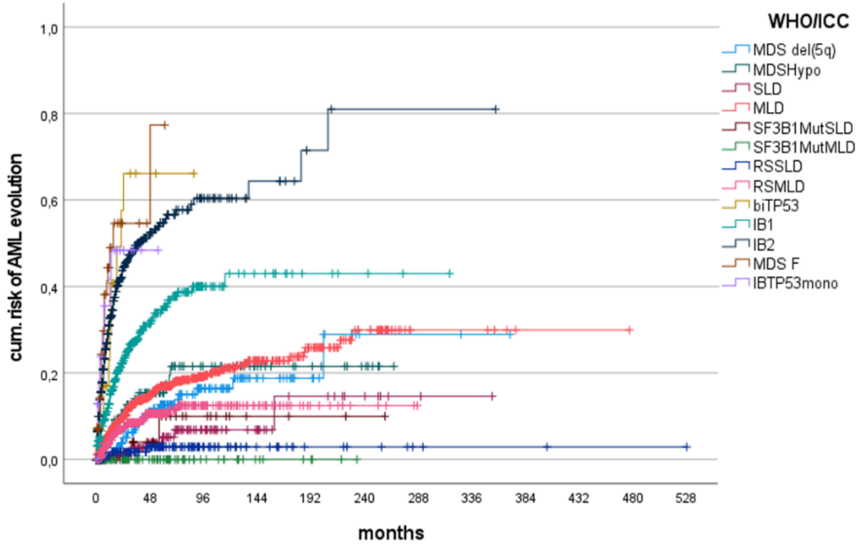

Supplement: Supplementary file 3 — Supplemental Figure 3 A-B [file 41375_2024_2157_MOESM3_ESM.pdf]
