# Supplementary material for: The new WHO 2022 and ICC proposals for the classification of myelodysplastic neoplasms. Validation based on the Düsseldorf MDS Registry and proposals for a merged classification
Source: Leukemia. 2024 Jan 23;38(2):442–5. doi: 10.1038/s41375-024-02157-2 (PMC10844089; doi:10.1038/s41375-024-02157-2)
Supplement: Supplementary file 4 — Supplemental Table 1 [file 41375_2024_2157_MOESM4_ESM.docx]

Supplemental Table 1: Detailed patients’ characteristics according to the WHO 2022 classification.

| **Parameter** | **All types** | **low blast count SLD** | **low blast count MLD** | **Hypopl.**  **MDS** | **MDS with SF3B1/RS** | **IB1** | **IB2** | **MDS**  **fibrosis** | **biallelic TP53 alteration** | **MDS del(5q)** | **MDS del(5q)**  **with TP53 mutation** | **MDS del(5q)**  **with SF3B1 mutation** | **AML MRC** |
| --- | --- | --- | --- | --- | --- | --- | --- | --- | --- | --- | --- | --- | --- |
|  | 5700 (100%) | 205  (3.6%) | 1775  (31.1%) | 143  (2.5%) | 768 (13.5%) | 850 (15%) | 914  (16%) | 80 (1.4%) | 41 (0.7%) | 207 (3.6%) | 8 (0.1%) | 19 (0.3%) | 690 (12.1%) |
| Gender m/f (%) | 56/44 | 57/43 | 58/42 | 51/49 | 54/46 | 60/40 | 57/42 | 65/35 | 54/46 | **27/73** | 50/50 | 53/47 | 55/45 |
| Post-cytotoxic MDN (%) | 6.2 | 3.4 | 4.6 | 10.5 | 5.7 | 6.1 | 8.7 | **12.6** | **12.2** | 4.9 | 0 | 10.5 | 6.8 |
| Age at diagnosis (median, range) | 71  18-104 | 73  32-91 | 72  18-99 | 68  18-93 | 72  18-92 | 70  20-104 | 70  18-96 | **65**  **32-86**  **p<0.001** | 66  50-95 | **67**  **21-90**  **p=0.025** | 65  56-72 | 75  53-85 | **72**  **53-85**  **p<0.001** |
| Hb g/dl (median, range) | 9,4  0-17,5 | 9,4  3,4-15,9 | 9,6  0-16,6 | 9,5  3,9-14,8 | **9,3**  **2,2-13,8**  **p=0.01** | 9,6  3,6-17,5 | 9,3  0-16,1 | **8,2**  **4,3-13,1**  **p<0.001** | 8,8  6-12,3 | **9**  **4.5-13,3**  **p=0.002** | 7,2  6-12,5 | 9,8  6-12,6 | **9,8**  **4-16**  **p=0.01** |
| Platelets x 1 000/µl (median, range) | 128  0-1540 | 166  5-709 | 128  0-1540 | **78**  **2-673**  **p<0.001** | 223  59-445 | **109**  **0-1408**  **p=0.01** | **85**  **3-1332**  **p=0.001** | **70**  **4-426**  **p<0.001** | **69**  **6-742**  **p=0.02** | **260**  **7-843**  **p=0.001** | 215  117-530 | 229  65-674 | **70**  **1-770**  **p<0.001** |
| WBC x 1 000/µl (median, range) | 3.8  0.1-48 | 4.4  1-19 | 4  3.2-48 | 3.3  0.3-18.5 | 4.9  0.5-28 | **3.4**  **0.2-19**  **p=0.01** | **3**  **2.2-26**  **p=0.001** | 3.4  0.6-26 | 3.4  1.1-22 | 4  1.4-15 | 4.1  2.5-11.2 | 5.3  2.6-16 | 2.9  0.1-48 |
| ANC x 1 000/µl (median, range) | 1.9  0.1-42 | 2.2  2-15 | 2.1  0.2-44 | 1.7  0.2-14 | 2.5  0.3-24 | **1.4**  **1-18**  **p=0.01** | **1.2**  **0.3-24**  **p=0.001** | 1.1  0.5-17 | **0.9**  **0.7-12**  **p=0.01** | 2.1  0.4-13 | 2.1  1.3-9 | 2.7  1.1-8.1 | **0.9**  **0.1-42**  **p<0.001** |
| Monocytes/µl (median, range) | 180  0-3400 | 335  0-980 | 213  0-920 | **140**  **0-828**  **p=0.02** | 242  0-980 | 163  0-962 | 120  0-994 | 94  11-945 | 154  57-696 | 210  0-935 | 63  25-112 | 307  0-980 | **92**  **0-3400**  **p<0.001** |
| Peripheral blast (%) (median, range) | 0  0-19 | 0  0-1 | 0  0-1 | 0  0-1 | 0  0-1 | 0  0-4 | 1  0-19 | 2  0-19 | 2  0-10 | 0  0-1 | 0 | 0  0-1 | 2  0-29 |
| Presence of peripheral blasts (%) | 25.2 | 8.2 | 6.2 | 7.8 | 6,3 | 35.4 | 50 | 62.5 | 67 | 7.9 | 0 | 26.7 | 58 |
| Median medullary blasts % (range) | 4  0-29 | 1  0-4 | 2  0-4 | 2  0-4 | 2  0-4 | 7  1-9 | **14**  **1-19**  **p<0.001** | **12**  **5-19**  **p<0.001** | **9**  **1-19**  **p=0.001** | 2  0-4 | 1  0-4 | 2  0-4 | **25**  **10-29**  **p<0.001** |
| Presence of ringsideroblasts (%) | 39.2 | 16.2 | 21.9 | 15 | 98.6 | 27.1 | 30.2 | 43.1 | 0 | 17 | 100 | **93.3** | 32.3 |
| SF3B1 mutation (%) | 48,2 | 0 | 0 | 0 | 88 | 27 | 18.5 | 0 | 0 | 0 | 33,3 | 71.4 | 25 |
| Percentage of ringsideroblasts (median, range) | 0  0-99 | 0  0-13 | 0  0-14 | 0  0-12 | 35  0-99 | 0  0-96 | 0  0-85 | 1  0-58 | 12  0-80 | 0  0-20 | 3  0-24 | **42.5**  **0-90** | 0  0-90 |
| Auer rods present (%) | 3.5 | 0 | 0 | 0 | 0 | 0 | 7.2 | 5.1 | **16.7** | 0 | 0 | 0 | 13.8 |
| abnormal karyotype % | 54.3 | 36.1 | 45.6 | 51.7 | 35.8 | 54.2 | 55.1 | 65.9 | **92.5** | **100** | 100 | 100 | 60.4 |
| complex karyotype % | 16.8 | 6 | 12.3 | 13.7 | 7.9 | 16.2 | 22.9 | **45.5** | **90** | 0 | 0 | 0 | 26.1 |
| del(5q) isolated  present % | 8.6 | 0 | 0 | 0 | 0 | 4,1 | 1,5 | 0 | 0 | 100 | 100 | 100 | 1.9 |
| Presence of TP53 mutation (%) | 20.4 | 9.4 | 10 | 9.1 | 12.2 | 11,1 | 29.5 | 75 | **100** | 0 | **100** | 0 | 12 |
| Chromosomal category according to IPSS-R (vl/low/int/high/vh) % | 4/56/18/  8/14 | 7/66/23/  4/5 | 7/57/20/  8/8 | 4/55/23/  8/10 | 4/68/13/  10/5 | 2/55/20/  9/13 | 2/50/18/  9/21 | **2/34/16/**  **5/43** | **0/7.5/0/**  **7.5/85** | 0/100/0/  0/0 | 0/100/0/  0/0 | 0/100/0/  0/0 | 2/44/21/  9/24 |
| Percentage of AML evolution (%) | 20 | 5 | 12 | 13 | 7 | 20 | **32** | **35** | **35** | 10 | 0 | 26 | n. a. |
| Cumulative AML evolution after 2 and 5 years (%) | 15/29 | 4/8 | 10/17 | 8/16 | 12/34 | 23/35 | 32/52 | 34/66 | 25/53 | 4/12 | n. a. | 12/34 | n. a. |
| Median overall survival in months (range) | 30  1-528 | 69  1-290 | 42  1-477 | 51  1-266 | 63  1-528 | 24  1-316 | 15  1-357 | 10  1-61 | 11  1-109 | 73  1-370 | 63  47-199 | 52  16-136 | 9  1-206 |

Continuous variables: Light and dark orange colored boxes indicate subgroups with significantly different median parameters compared to the concurring subgroups.

Categorial variables: Dark orange colored boxes indicate subgroups with dichotomic parameters that are significantly different to the rest (p<0.001).
