# Supplementary material for: The new WHO 2022 and ICC proposals for the classification of myelodysplastic neoplasms. Validation based on the Düsseldorf MDS Registry and proposals for a merged classification
Source: Leukemia. 2024 Jan 23;38(2):442–5. doi: 10.1038/s41375-024-02157-2 (PMC10844089; doi:10.1038/s41375-024-02157-2)
Supplement: Supplementary file 5 — Supplemental Table 2 [file 41375_2024_2157_MOESM5_ESM.docx]

Supplemental Table 2: Prognostic parameters (entire population)

Parameter median survival in months χ2 p Progression to AML (%) χ2 p

ANC

<800 16 85.3 <0.0005 17.8 205.1 <0.00005

>800 31 34.4

Platelets

>100 41 285.5 <0.00005 16.2 172.5 <0.00005

50-100 21 25.4

<50 12 25.8

Hb

>10 44 212.6 <0.00005 18.4 43.4 <0.00005

8-10 25 20.6

<8 15 22.8

Karyotype risk groups according to IPSS-R

Very low 67 415.8 <0.00005 8.1 331.9 <0.00005

Low 53 19.0

Intermediate 28 30.2

High 24 34.2

Very high 10 45.1

Myelofibrosis

Grade 0-1 32 25.2 <0.00005 20.5 10.1 0.002

Grade 2-3 17 26.9

TP53 status

Mutated 24 31.9 <0.00005 29.2 28.2 <0.00005

WT 70 12.2

TP53 alteration

Bialleic 11 40.9 <0.00005 40.7 29.5 <0.00005

Not biallelic 63 14.1

Marrow blast percentage

<2% 51 637.6 <0.00005 7.3 1390.1 <0.00005

3-4% 37 14.7

5-10% 20 21.4

10-19% 14 32.5

Grade of dysplasia

Unilineage 70 75.8 <0.00005 4 79.4 <0.00005

Multilineage 27 20

Peripheral blasts

No 33 144.2 <0.00005 16.2 303.1 <0.00005

Yes 14 35.2
