# Supplementary material for: The new WHO 2022 and ICC proposals for the classification of myelodysplastic neoplasms. Validation based on the Düsseldorf MDS Registry and proposals for a merged classification
Source: Leukemia. 2024 Jan 23;38(2):442–5. doi: 10.1038/s41375-024-02157-2 (PMC10844089; doi:10.1038/s41375-024-02157-2)
Supplement: Supplementary file 6 — Supplemental Table 3 [file 41375_2024_2157_MOESM6_ESM.docx]

Supplemental Table 3: Multivariate analyses with regard to overall survival

a) only patients in whom information on following parameters were available at diagnosis: medullary blast count, cellularity, fibrosis, karyotype according to the IPSS-R, TP53 mutation status, presence of multilineage dysplasia, cell counts (n=141)

Variable entered into the model χ2 p RR

TP53 mutated 11.1 0.001 2.98

Presence of multilineage dysplasia 3.8 0.048 1.5

b) patients with information on medullary blast count, karyotype according to the IPSSR, TP53 mutation status, presence of multilineage dysplasia, (n=412)

Variable entered into the model χ2 p RR

TP53 mutated 13.97 <0.0005 2.1

marrow blasts percentage (cont. variable) 11.4 0.001 1.1

karyotype according to IPSS-R 10.2 0.001 1.3
