# Supplementary material for: The new WHO 2022 and ICC proposals for the classification of myelodysplastic neoplasms. Validation based on the Düsseldorf MDS Registry and proposals for a merged classification
Source: Leukemia. 2024 Jan 23;38(2):442–5. doi: 10.1038/s41375-024-02157-2 (PMC10844089; doi:10.1038/s41375-024-02157-2)
Supplement: Supplementary file 7 — Supplemental Table 4 [file 41375_2024_2157_MOESM7_ESM.docx]

Supplemental Table 4: Detailed patients’ characteristics according to the ICC classification.

| **Parameter** | **All types** | **NOS with SLD** | **NOS with MLD** | **MDS with SF3B1** | **MDS del(5q)** | **MDS with TP53** | **MDS/AML with TP53** | **MDS EB** | **MDS AML** |
| --- | --- | --- | --- | --- | --- | --- | --- | --- | --- |
|  | 5700  (100%) | 378  (6.6%) | 2360  (41.2%) | 123  (2.2%) | 234  (4.1%) | 59  (1.1%) | 36  (0.6%) | 878  (15.3%) | 932  (16.6%) |
| Gender m/f (%) | 56/44 | 51/49 | 57/43 | 57/43 | **30/70** | 55/45 | 67/33 | 60/40 | 58/42 |
| Post-cytotoxic MDN (%) | 6.2 | 4.2 | 4.7 | 1.6 | 4.3 | 8.1 | **11.1** | 5.1 | 7.3 |
| Age at diagnosis (median, range) | 71  13-104 | 72  16-91 | 72  13-100 | 68  30-85 | 69  21-90 | 69  29-85 | 69  19-95 | 70  20-104 | 70  16-96 |
| Hb g/dl (median, range) | 9.4  0-17.5 | 9.5  3.4-15.9 | 9.5  0-16.6 | 9.6  6.5-13.5 | **9**  **4.5-13.3**  **p=0.002** | 9.9  6.7-13.3 | **8.9**  **6-12.5**  **p=0.001** | 9.6  2.6-17.5 | 9.3  3.1-16.1 |
| Platelets x 1.000/µl (median, range) | 116  1-1540 | 186  5-709 | 138  0-1540 | 248  12-444 | **252**  **7-843**  **p=0.001** | 97  19-742 | **71**  **6-354**  **p=0.04** | 101  1-1408 | **80**  **3-1121**  **p<0.001** |
| WBC x 1000/µl (median, range) | 3.7  0.1-269 | 4.8  1-19 | 4  3.2-68 | 4.8  0.5-75 | 4  1.4-150 | **3**  **1.2-12.2**  **p=0.03** | 3.3  1.3-22.1 | 3.3  0-109 | **2.9**  **0.2-268**  **p<0.001** |
| ANC x 1000/µl (median, range) | 1.8  0.1-229 | 2.7  0.2-52 | 2.2  0-92.3 | 2.4  0.3-59 | 2.1  0.4-13.5 | **1.2**  **1-4.4**  **p=0.006** | 1.4  0.8-12 | 1.5  0-52 | **1.2**  **0-51**  **p=0.004** |
| Monocytes/µl (median, range) | 182  0-32648 | 295  0-3078 | 207  0-4774 | 360  0.4-1748 | 213  0-1050 | 168  0-620 | 296  0-848 | 156  0-4200 | **111**  **0-3060**  **p=0.002** |
| Peripheral blast (%) (median, range) | 0 (0-19) | 0 (0-1) | 0 (0-1) | 0 (0-1) | 0 (0-1) | 0 (0-5) | 2.5 (0-10) | 0 (0-8) | 1 (0-19) |
| Presence of peripheral blasts (%) | 21.1 | 6.6 | 6.2 | 13.1 | 9.8 | **34.6** | **93.7** | **36.3** | **50.4** |
| Median medullary blasts % (range) | 4 (0-95) | 2 (0-4) | 2 (0-4) | 2 (0-4) | 2 (0-4) | **4 (0-9)**  **p=0.001** | **15 (5-19)**  **p<0.001** | **7 (0-9)**  **p=0.001** | **14 (0-19)**  **p<0.001** |
| Presence of Ringsideroblasts (RS) (%) | 25.4 | 59.2 | 21.9 | **93.3** | 33 | 52.6 | 66.7 | 30.3 | 35.8 |
| SF3B1 mutation (%) | 44.6 | 0 | 0 | 100 | 40 | 29.2 | 40 | 26.2 | 13 |
| Percentage of RS (median, range) | 0 (0-99) | 15 (0-97) | 0 (0-99) | 20 (0-99) | 0 (0-90) | 5 (0-80) | 8 (0-26) | 0 (0-96) | 0 (0-85) |
| Auer rods present (%) | 3.6 | 0 | 0 | 0 | 0 | 0 | **9.1** | 0.2 | **10.1** |
| abnormal karyotype % | 54.3 | 36.7 | 45.5 | 20.2 | **100** | **77.4** | **91.7** | 57.8 | 54.5 |
| complex karyotype % | 16.8 | 5.4 | 12.2 | 1.9 | 0 | **54.8** | **77.8** | 15.4 | 22.8 |
| del(5q) isolated present % | 8.5 | 0 | 0 | 0 | **100** | 1.6 | 5.6 | 6.4 | 2.7 |
| Presence of TP53 mutation (%) | 21.9 | 0 | 0 | 0 | 23.5 | **100** | **100** | 1.2 | 0 |
| Chromosomal category according to IPSSR (vl/low/int/high/vh) % | 4/59/19/  6/14 | 6/61/18/  2/4 | 6/57/20/  7/9 | 0/81/11/  5/4 | 0/100/0/  0/0 | **3/30/5/**  **16/46** | **0//10/10**  **14/66** | 2/54/22/  7/15 | 2/51/19/  6/21 |
| Percentage of AML evolution (%) | 20.4 | 4.7 | 10.9 | 3.9 | 11.2 | 25.8 | **36.1**  **p<0.001** | **20.7**  **p<0.001** | **31.6**  **p<0.001** |
| Cumulative AML-evol. after 2/ 5 years (%) | 15/29 | 3/7 | 10/16 | 3/5 | 5/14 | 29/34 | 56/56 | 24/37 | 44/54 |
| Median overall survival in months (range) | 30  0-528 | 68  0-528 | 41  0-520 | 126  0-258 | 75  0-370 | 24  0-116 | 16  0-76 | 22  0-316 | 14  0-405 |

Continuous variables: Light and dark orange colored boxes indicate subgroups with significantly different median parameters compared to the concurring subgroups.

Categorial variables: Dark orange colored boxes indicate subgroups with dichotomic parameters that are significantly different to the rest (p<0.001).
